# Multicentre phase II studies evaluating imatinib plus hydroxyurea in patients with progressive glioblastoma

**DOI:** 10.1038/sj.bjc.6605411

**Published:** 2009-11-10

**Authors:** D A Reardon, G Dresemann, S Taillibert, M Campone, M van den Bent, P Clement, E Blomquist, L Gordower, H Schultz, J Raizer, P Hau, J Easaw, M Gil, J Tonn, A Gijtenbeek, U Schlegel, P Bergstrom, S Green, A Weir, Z Nikolova

**Affiliations:** 1 The Preston Robert Tisch Brain Tumor Center, Duke University Medical Center, Durham, NC, USA; 2 Zentrum für Neuro-Onkologie am Ärztehaus Velen, Velen, Germany; 3 Hopital Pitie Salpétrière, Paris, France; 4 Centre René Gauducheau, Saint-Herblain, France; 5 Daniel den Hoed Cancer Center, Erasmus University Hospital, Rotterdam, The Netherlands; 6 UZ Gasthuisberg, Leuven, Belgium; 7 Onkologikliniken Akademiska sjukhuset, Uppsala, Sweden; 8 CHU Erasme, Brussels, Belgium; 9 Aarhus University Hospital, Århus, Denmark; 10 Northwestern University, Feinberg School of Medicine, Northwestern Memorial Hospital, Chicago, IL, USA; 11 Klinik und Poliklinik für Neurologie der Universität Regensburg, Universitaetsklinikum Regensburg, Regensburg, Germany; 12 Tom Baker Cancer Center, Calgary, Alberta, Canada; 13 Institut Català d’Oncologia, Hospital Durans I Reynals, L’Hospitalet de Llobregat, Barcelona, Spain; 14 LMU München, München, Germany; 15 Radboud University Nijmegen Medical Centre, Nijmegen, The Netherlands; 16 Knappschaftskrankenhaus, Ruhr-University Bochum, Germany; 17 Onkologikliniken, Norrlands Universitetssjukhus, Umea, Sweden; 18 Novartis Pharma AG, Basel, Switzerland

**Keywords:** glioblastoma, imatinib mesylate, platelet-derived growth factor, c-KIT

## Abstract

**Background:**

We evaluated the efficacy of imatinib mesylate in addition to hydroxyurea in patients with recurrent glioblastoma (GBM) who were either on or not on enzyme-inducing anti-epileptic drugs (EIAEDs).

**Methods:**

A total of 231 patients with GBM at first recurrence from 21 institutions in 10 countries were enrolled. All patients received 500 mg of hydroxyurea twice a day. Imatinib was administered at 600 mg per day for patients not on EIAEDs and at 500 mg twice a day if on EIAEDs. The primary end point was radiographic response rate and secondary end points were safety, progression-free survival at 6 months (PFS-6), and overall survival (OS).

**Results:**

The radiographic response rate after centralised review was 3.4%. Progression-free survival at 6 months and median OS were 10.6% and 26.0 weeks, respectively. Outcome did not appear to differ based on EIAED status. The most common grade 3 or greater adverse events were fatigue (7%), neutropaenia (7%), and thrombocytopaenia (7%).

**Conclusions:**

Imatinib in addition to hydroxyurea was well tolerated among patients with recurrent GBM but did not show clinically meaningful anti-tumour activity.

There are currently few effective treatment options available for adults with glioblastoma (GBM), the most common malignant primary brain tumour. Median survival for newly diagnosed GBM is approximately 15 months from diagnosis, after standard therapy of temozolomide (TMZ) and radiotherapy and only 26% of patients survive 2 years (Stupp *et al*, 2005). Although recent studies with therapeutics targeting vascular endothelial growth factor (VEGF) have shown encouraging results (Vredenburgh *et al*, 2007; Friedman *et al*, 2009), most salvage therapies after progression have proven ineffective, with a median time to progression of only 9 weeks, low response rates and life expectancy of only a few months (Wong *et al*, 1999; Ballman *et al*, 2007; Lamborn *et al*, 2008).

Imatinib mesylate (Glivec or Gleevec), a tyrosine kinase inhibitor of platelet-derived growth factor receptors (PDGFRs) *α* and *β;* c-KIT, the receptor for stem cell factor; c-Fms, the receptor for macrophage-colony stimulating factor; Abl, and Arg TK, is currently approved for several indications including Philadelphia chromosome-positive (Ph+) chronic myelogenous leukaemia, Ph+ acute lymphoblastic leukaemia, KIT (CD 117)-positive, unresectable or metastatic malignant gastrointestinal stromal tumours, and four rare diseases (hypereosinophilic syndrome, dermatofibrosarcoma protuberans, myelodysplastic/myeloproliferative diseases, and systemic mastocytosis).

Several factors suggest that imatinib may be an active therapeutic for malignant glioma. First, gliomas frequently overexpress PDGF and PDGFRs in an autocrine/paracrine manner (Nister *et al*, 1988; Hermanson *et al*, 1992; Guha *et al*, 1995; Lokker *et al*, 2002). Second, c-KIT is expressed by many GBM tumours (Went *et al*, 2004; Joensuu *et al*, 2005). Third, imatinib inhibits the growth of human GBM cell lines and prolongs survival of nude mice with intracranial GBM cell implants (Kilic *et al*, 2000), whereas expression of PDGFR and the chemokine CXCL12/SDF-1 (stromal cell-derived factor-1) predict imatinib sensitivity *in vitro* (Hagerstrand *et al*, 2006). Finally, imatinib can sensitise human GBM cells to ionising radiation (Russell *et al*, 2003; Holdhoff *et al*, 2005).

Preclinical evidence suggests that imatinib may enhance chemosensitivity by several possible mechanisms, including decreasing interstitial fluid pressure (Heuchel *et al*, 1999; Pietras *et al*, 2001, 2002, 2003; Ostman, 2004), decreasing angiogenesis (Bergers *et al*, 2003; Dudley *et al*, 2003; Hwang *et al*, 2003; Uehara *et al*, 2003; Apte *et al*, 2004; Pietras and Hanahan, 2005), and by affecting DNA repair mechanisms (Aloyz *et al*, 2004), stromal chemokines (Ostman, 2004), and multidrug transporter activity (Houghton *et al*, 2004; Ozvegy-Laczka *et al*, 2004). However, the uptake and distribution of imatinib in the brain is limited by P-gp/BCRP-mediated efflux (Dai *et al*, 2003), suggesting that combination regimens with imatinib may be more effective than imatinib monotherapy. Clinical studies evaluating imatinib as monotherapy in relapsed GBM showed response rates ranging from 3 to 6% and a progression-free survival at 6 months (PFS-6) rate of 16% (Wen *et al*, 2006; Raymond *et al*, 2008).

Hydroxyurea (HU) has been used in multiagent regimens against GBM, medulloblastoma, and metastatic brain tumours (Levin and Prados, 1992; Geyer *et al*, 1994; Kyritsis *et al*, 1996; Kaba *et al*, 1997; Prados *et al*, 1998). Hydroxyurea exerts a cell-cycle-specific effect during early S-phase, through inhibition of ribonucleotide reductase, thereby blocking DNA synthesis (Kyritsis *et al*, 1996). Hydroxyurea exhibits rapid absorption from the gastrointestinal tract after oral dosing and readily crosses the blood–brain barrier. Independently conducted, single-institutional clinical studies have noted anti-tumour activity among patients with recurrent grade 3 (Desjardins *et al*, 2007) and 4 malignant glioma (Dresemann, 2005; Reardon *et al*, 2005) after treatment with imatinib (400–1000 mg daily) and HU (1000 mg daily). Each of these studies also confirmed that this combination was well tolerated.

Enzyme-inducing anticonvulsant drugs (EIACDs; eg phenytoin, carbamazepine, phosphenytoin, oxcarbamazepine, and phenobarbital) are known to accelerate the metabolism of imatinib, resulting in a shorter plasma-elimination half-life (*t*_1/2_) and a lower area under the plasma concentration time curve (AUC) (Reardon *et al*, 2005; Wen *et al*, 2006). We sought to further evaluate the efficacy and tolerability of imatinib in addition to HU among patients with recurrent GBM treated on multi-institutional phase II studies depending on the use of EIACDs or not. Specifically, for study H2201, patients were not on EIACDs, whereas study H2202 only included patients on EIACDs. The primary end point was objective overall response rate (ORR); secondary end points were safety, overall survival (OS), and PFS.

## Patients and methods

### Study design and treatment

We conducted two, parallel open-label, multicentre, single-arm, phase II trials. In study H2201, patients were not allowed to be on EIACDs whereas patients were enrolled on study H2202 if they were on EIACDs. The dose of imatinib differed between the trials to account for the effect of EIACDs on imatinib metabolism (Reardon *et al*, 2005; Wen *et al*, 2006): Patients enrolled on study H2201 received 600 mg once a day, whereas those on study H2202 received 500 mg twice a day. For both trials, patients received 500 mg of HU twice a day. The studies were identical in all other respects. Medically appropriate efforts were used to maintain study-specific EIACD exposure for patients on each study; however, H2201 patients were deemed off study if they initiated EIACDs and H2202 patients were similarly censored if they discontinued EIACDs.

Patients remained on study unless they withdrew consent, developed tumour progression or unacceptable toxicity. During the first year of treatment, patients were assessed every 4 weeks, changing to every 8 weeks thereafter. The study was designed according to respective national regulations and was approved by local ethical review boards before patient accrual started. All patients provided written informed consent according to local and national regulations.

### Patient eligibility

All patients were required to have institutional histological confirmation of GBM that was at first recurrence after conventional therapy and measurable disease on gadolinium-enhanced MRI. After study entry, centralised review was conducted to confirm histopathology. Satisfactory haematologic (haemoglobin ⩾10 g per 100 ml, absolute neutrophil count >1500 cells per litre, platelets >100 000 cells per litre), biochemical (serum creatinine <1.5 mg per 100 ml, BUN <25 mg per 100 ml; AST and bilirubin <1.5 × upper limit of normal) and performance status (ECOG score ⩽2 or Karnofsky ⩾60%) parameters were also required.

Key exclusion criteria included peripheral oedema ⩾grade 2; pulmonary, pericardial or peritoneal effusions of any grade; an excessive risk of an intracranial haemorrhage; major surgery within 2 weeks before study entry; and concurrent warfarin administration.

### Patient assessments

Disease status was assessed using a modified version of the Macdonald criteria (Macdonald *et al*, 1990), including an increase in corticosteroid dosing, regardless of radiographic or clinical assessment, as a criteria to define progressive disease (PD). Sites performed each assessment, before submitting the data to a central independent review (CIR) team. The CIR team included three radiologists (two reviewers and an adjudicator) as well as a neuro-oncologist who conducted a two-stage evaluation of each patient assessment. First, steroid and MRI assessments were independently determined and included in the database. Second, the site neurologic assessment was incorporated to provide an overall outcome assessment. Visits were assessed in sequence; older visits could be reviewed, but newer visits could only be seen after completion of the preceding visit. Adjudication occurred if the overall visit conclusion differed between the two radiologists. To minimise bias, all CIR team members were blinded to the patient study identifier, the results of the other reader, and the final outcome of the patient. Final outcome was hidden by presenting data of each time point only after the previous time point had been assessed.

Safety assessments included weekly complete blood counts and monthly chemistry profiles. Treatment was held until any non-haematologic grade 3 or 4 event resolved to grade ⩽1, after which the study regimen was resumed with a reduction in daily imatinib dosing by 200 mg. If the event recurred, HU was reduced to 500 mg a day. If the event recurred despite these dose modifications, patients were taken off study. The above guidelines were also followed for grade 3 or greater thrombocytopaenia and grade 4 neutropaenia. Myeloid and erythoid growth factors were permitted according to established guidelines.

### Tumour biomarker analysis

Archival tumour samples from either initial diagnosis or after prior therapy failure were analysed at the Duke University Hospital Cell Imaging Laboratory (Durham, NC, USA). Methylguanine methyltransferase (MGMT), PDGFR*α* and PDGFR*β*, phospho-p44/42 MAPK, phospho-S6 ribosomal protein, phospho-AKT, PTEN, EGFR, and EGFRvIII were assessed using immunohistochemistry (IHC) reagents and methods (Supplementary Methods). Similarly, dual-colour fluorescent *in situ* hybridisation (FISH) was performed on formalin-fixed, paraffin-embedded tissue specimens using commercially available probes, including EGFR/CEP 7, PTEN/CEP 10 (Vysis, Downers Grove, IL, USA), and c-KIT/CEP 4 for EGFR, PTEN, and c-KIT DNA locus copy number (Supplementary Methods). Genes were classified as polysomic or amplified if their copy number relative to respective centromere probe exceeded 1 or 2, respectively.

### Pharmacokinetic analysis

Blood samples were collected from a subset of patients on each study on days 6 and 29 before treatment and 0.5, 1, 2, 3, 4, 8, 12, 13, 14, and 24 h after their morning dose. For these patients, HU was initiated after the 24-h sample for day 6 was obtained. Plasma supernatants were separated by centrifugation and immediately frozen (−20°C). The day 29 plasma supernatants were split for both imatinib and HU analyses. Plasma concentrations of imatinib and its metabolite, CGP74588, were determined by high-pressure liquid chromatography/mass spectrometry (Parise *et al*, 2003) whereas HU plasma concentrations were measured by gas chromatography and mass spectroscopy. The individual plasma concentration of data for each subject was used to calculate pharmacokinetic parameters according to the model-independent approach using WinNonlin software (Version 5.2; Pharsight Corporation, Mountain View, CA, USA). Nominal sampling times were used for calculating summary statistics of the plasma concentration data.

### Statistical considerations

A total of 220 adult patients (110 in each study) were planned to enroll. Wong *et al* (1999) previously reported a 5% radiographic response rate among patients with GBM treated with TMZ at first recurrence. In the current studies, 110 patients were estimated as required per study to provide a 95%, two-sided confidence interval for radiographic response to have a lower limit of 5%, including a 10% expected dropout rate.

An interim analysis was performed after 101 patients were enrolled in both studies combined. Accrual continued until a data cut-off occurred 4 months after the 101st patient had been enrolled to allow for at least 16 weeks of treatment. At the interim analysis, if there were ⩽4 responses (CR or PR) observed from 101 patients, both studies were to be terminated due to lack of efficacy. If the studies continued, combination therapy was to be declared as having insufficient activity if there were ⩽15 responses observed from the 220 patients to be recruited.

A log-rank test was used to compare the OS and PFS experience of patient subgroups defined by various biomarkers. Patients with inadequate samples to analyse for a particular marker were excluded from that specific statistical analysis. Within subgroups, the Kaplan–Meier estimator was used to generate estimates of median OS and PFS, as well as 6-, 12-, and 24-month OS and PFS rates.

## Results

### Patient characteristics

The intent-to-treat (ITT) population consisted of 231 enrolled patients including 131 on study H2201 and 100 on study H2202. Study H2201 accrued more rapidly, hence a higher percentage of eligible patients were not on EIACDs. Otherwise, characteristics of patients accrued to both studies were comparable (Table 1). Adequate tumour material for central histopathology review was available from 177 patients (77.7%) and confirmed GBM in 158 cases (89.3%). This rate is comparable to that previously reported in other multi-institutional studies (Stupp *et al*, 2005; Raymond *et al*, 2008). The remaining patients had either grade 3 malignant glioma (*n*=11, 7.0%), grade 2 glioma (*n*=2, 1.3%), or were non-diagnostic (*n*=6, 3.8%). Most patients (88.7%) had just one measurable lesion at baseline. Only 11.7% of patients had additional lesions documented for evaluation, which were too small to measure (‘evaluable lesions’). According to the CIR, 9 patients (3.9%) had only evaluable lesions at their baseline MRI scan. Thirty-three patients (14%) enrolled within 3 months of XRT/daily TMZ completion; however, outcome among this subset did not differ compared to the remaining patients (data not shown) indicating that enrolment of patients with possible pseudoprogression (Brandes *et al*, 2008; Brandsma *et al*, 2008) did not influence the outcome of this study. No patients had to discontinue study participation due to changing enzyme-inducing anti-epileptic drug (EIAED) status.

### Toxicity

The median times on study were 6 weeks (range, 1.3–91 weeks) and 4 weeks (range, 0.1–102 weeks) for study H2201 and H2202, respectively. A total of 195 patients (84.8%) received less than 180 days of therapy whereas 20 patients (8.7%) and 15 patients (6.5%) remained on study for 180–365 days and >365 days, respectively.

The adverse events seen in the study were as expected for this population and these agents. In general, they were mild and transient (Table 2). There were no major differences in adverse events between the two studies. Dose adjustment or interruption due to adverse events affected 34% of H2201 patients and 39% of H2202 patients. Patients on each study (8%) discontinued therapy due to toxicity. Among grade 3 or higher events, the most common included fatigue (7.0%), neutropaenia (6.9%), and thrombocytopaenia (6.9%). There were no grade 5 attributable to adverse events.

### Pharmacokinetic analyses

Population PK samples were obtained from 15 patients on study H2201 and 6 patients on study H2202. Overall, imatinib, CGP74588, and HU pharmacokinetic results were consistent with those previously reported and confirm the marked impact of EIAEDs on imatinib metabolism (Table 3; Reardon *et al*, 2005). Specifically, dose-normalised Cmax and AUCs of imatinib were lowered for patients on EIAEDs compared with those who were not on EIAEDs. Slight elevations of CGP74588 compared with parent drug ratio were noted for patients on EIAEDs (0.23 *vs* 0.19) or not receiving EIAEDs (0.41 *vs* 0.35) in the presence of HU. Imatinib exposure on day 29 (with HU) was slightly increased compared with day 6 (imatinib alone) for patients on study H2201, but was slightly decreased for patients on study H2202, although these differences did not achieve statistical significance. The exposure and elimination of HU were not different between patients receiving or not receiving EIAEDs.

### Tumour biomarker analysis

**Figure 1:**
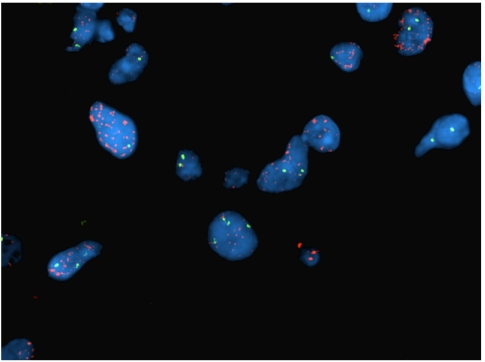
Representative example of c-KIT gene amplification detected by fluorescence *in situ* hybridisation (FISH). High-level amplification of c-KIT (red signals) detected along with two copies of chromosome 4 centromeres (green signals).

Table 4 summarises the number of patients who had sufficient archival tumour material available for biomarker analysis by IHC and FISH, as well as the association of marker expression with outcome. Of note, 80 of 91 (88%) assessable tumours were positive for PDGFR*α*, whereas 47 of 67 (70%) assessable tumours expressed PDGFR*β*. Among 25 tumours that were assessable for c-KIT copy number analysis by FISH, 1 (4%) had evidence of gene amplification (Figure 1) and 1 (4%) was polysomic.

### Outcome

Table 5 shows the study analysis populations. The safety population was one less than the ITT population because one patient was lost to follow-up immediately after baseline assessments. At the time of data analysis, most patients were off study with disease progression or death (82%), whereas adverse events, withdrawal of consent, completion of planned therapy, and miscellaneous factors accounted for 8, 5, 3, and 3% of study discontinuations, respectively.

Overall, five patients on study H2201 (3.8%) and three on study H2202 (3%) achieved either a PR or CR confirmed by the CIR giving an ORR of 3.4% (Table 5). In addition, 19% of patients on each study achieved stable disease (SD). Of note, 13 patients (5.6%) were classified as CR or PR by local investigators, but were not confirmed by the CIR. Nineteen patients (8.2%) had either a confirmed CR or PR, or achieved SD lasting 6 months or more. With one confirmed responder and three unconfirmed responders at the interim analysis, a lack of efficacy for the drug combination was concluded as per the statistical design, and the trial was closed for accrual. However, accrual was sufficiently robust in the interval between enrolment of the 101st patient and completion of the interim analysis that study H2201 actually over-accrued, and study H2202 accrued 100 of the planned 110 patients.

**Figure 2:**
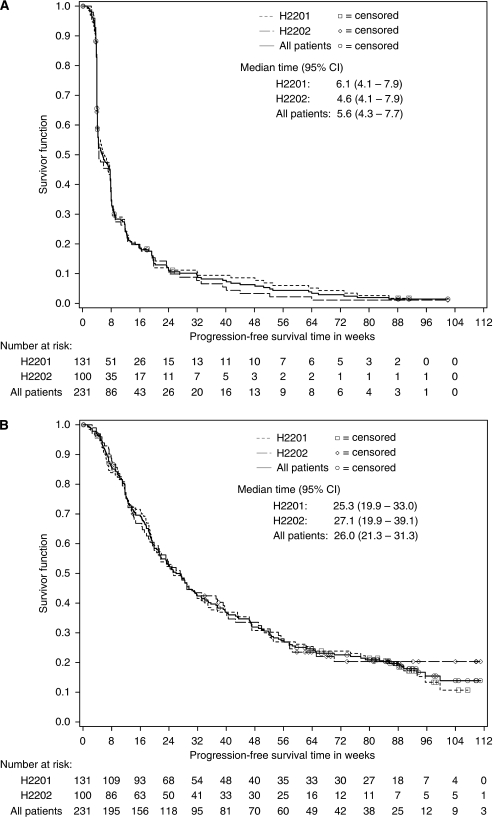
Kaplan–Meier plots of progression-free survival (**A**) and overall survival (**B**) for patients enrolled on H2201 and H2202 studies.

Progression-free survival rates at 6 months were 11.2% (95% CI, 5.7–16.6) for study H2201 and 9.9% (95% CI, 3.8-15.9) for study H2202 (Table 5; Figure 2A). Progressive disease was defined by MRI assessment in 114 patients (51.6%), whereas 41 patients (18.6%) were defined as progressive by increased corticosteroid use only and 26 patients (11.8%) solely by neurologic decline. Forty patients (18.1%) died, without previous PD determination, presumably due to PD. Median OS was 25.3 weeks (95% CI, 19.9–33.0) for study H2201 and 27.1 weeks (95% CI, 19.9–39.1) for study H2202 (Table 4; Figure 2B).

None of the tumour markers assessed by IHC among archival tumour material obtained from patients correlated with PFS (Table 4). However, single patients with either c-KIT amplification or polysomy remained progression-free for 290 and 232 days, respectively. Compared with patients with normal c-KIT copy number (*n*=23), PFS was increased among those with either c-KIT polysomy or amplification (*P*=0.021).

## Discussion

Two prior, single-institutional studies independently suggested that the combination of imatinib and HU had anti-tumour activity among patients with recurrent GBM. Dresemann (2005) first reported that 6 of 30 patients (20%) achieved a radiographic response whereas 11 additional patients (37%) achieved SD for a median of 6 months (range 3–32 months). In a follow-up phase II study among 33 patients, Reardon *et al* (2005) noted a radiographic response rate of 9% and PFS-6 of 27%. The current phase II studies were conducted to further evaluate imatinib in addition to HU in a multi-institutional setting. If the current multi-institutional studies achieved evidence of anti-tumour benefit comparable to that observed in the two prior single-institutional studies, a randomised, multicentre phase III study comparing imatinib in addition to HU to alternative salvage therapy was anticipated.

Toxicity in the current studies was similar to that reported previously confirming that imatinib in addition to HU is well tolerated among patients with recurrent GBM. The most common toxicities were haematologic and included neutropaenia (grade 3, *n*=12, 5%; grade 4, *n*=5, 1.7%) and thrombocytopaenia (grade 3, *n*=10, 4%; grade 4, *n*=6, 3%). The most common non-haematologic toxicities included nausea (39%), fatigue (34%), peripheral oedema (24%), and diarrhoea (19%), although the majority of these events were grade 2.

Outcome on the current studies was poorer than noted on the prior single-institutional studies, and the trials were closed after the interim analysis. Specifically, only 3.4% of patients achieved a confirmed radiographic response, and the PFS-6 was only 10.6%. Of note, we did not observe a significantly different outcome among patients treated on the study H2201 and H2202 trials. Several factors may have contributed to the discrepancy in outcome between the current multicentre studies and the previously reported single-institutional studies. First, single-centre studies may bias towards enrolment of more favourable patients. Approximately 28% of patients in the current studies received less than 25 days of treatment, typically progressing or dying within this 25-day period. Such patients probably had a poor prognosis and rapid disease advancement on entry to the trial, such that receipt of treatment may have been too late to control disease. In contrast, none of the patients reported by Reardon *et al* (2005) discontinued therapy prematurely due to rapidly progressive tumour. Imatinib achieves plasma steady-state concentrations after approximately 6 days, but additional time to achieve stable concentrations within the brain is likely required among patients with GBM due to the impact of the blood–brain barrier.

Second, incorporation of rigorous independent, blinded, centralised outcome review with strict assessment guidelines in the current studies may have impacted the rates of determined response (Dodd *et al*, 2008). In contrast, responses were assessed solely by study investigators in the prior single-centre studies.

Third, this study included an increase in corticosteroid dosing, independent of clinical status and radiologic findings, to define PD. The Macdonald criteria define PD based on ‘⩾25% increase in size of enhancing tumour or any new tumour on CT or MRI scans, or neurologically worse, and steroids stable or increased.’ In fact, the original publication stated, ‘Patients requiring escalating steroid doses to maintain neurologic function, in the absence of significant CT worsening (ie, <25% increase or no change) may have early tumor progression but are included in stable category’. Indeed, many clinicians do not interpret an increase in corticosteroids to be a sufficient ‘stand-alone’ criteria for defining PD because clear-cut guidelines to specify when an increase in corticosteroid dosing is appropriate for patients with primary brain tumour are not defined, and no cut-off has been specified for what change in dosage determines a meaningful ‘dose increase’. Furthermore, corticosteroids are routinely prescribed to patients with primary brain tumour to improve neurologic deterioration or alleviate increased cerebral oedema due to several factors independent of underlying tumour status, including overly aggressive corticosteroid tapering, seizures, post-operative sequellae, infection, metabolic disturbances, and vascular events. In general, though the decision to increase corticosteroids is individually made by the treating physician based on either worsened neurologic symptoms or radiographic findings such as increased oedema. Nonetheless, in the absence of co-morbid events or changes due to concurrent medications, the need for increased corticosteroids to treat neurologic deterioration is usually due to progressive tumour. Moreover, increasing steroid dosage may mask both clinical deterioration and increased enhancement qualifying for progression. We therefore chose to include this parameter in this study to increase the rigour of PD assessment.

Biologic factors underlying response to imatinib are not well characterised among patients with malignant glioma. In preclinical GBM studies, PDGFR and CXCL12/SDF-1 expression are associated with imatinib sensitivity (Hagerstrand *et al*, 2006). Although expression of CXCL12/SDF-1 was not assessed in this study, IHC expression of PDGFR did not correlate with PFS. However, we noted statistically increased PFS among two patients with either c-KIT gene amplification or polysomy compared with patients with normal c-KIT copy number. Clearly, these results must be interpreted cautiously due to the small number of patients. However, to further investigate this potential association, we evaluated c-KIT copy number among patients with available archival tumour material who were treated on a previously published phase 2 study (Reardon *et al*, 2005). Specifically, among 12 patients with adequate tumour material available for analysis, 3 had c-KIT polysomy or gene amplification. Although the differences did not reach statistical significance (*P*=0.15), the median PFS and PFS-6 were 16.7 weeks (95% CI, 8.3–130.3) and 40% (95% CI, 5.2–75.3%) for patients with increased c-KIT copy number compared with 8.5 weeks (95% CI, 7.9–16.1) and 10% (95% CI, 0.6–35.8) for those with normal copy number. Future studies to evaluate c-KIT gene copy number or protein expression as a biomarker of response to imatinib therapy in patients with recurrent GBM may be warranted.

Of note, Dresemann *et al* (2008) recently reported preliminary results of a clinical trial in which patients with recurrent GBM were randomised to receive either imatinib and HU *vs* HU alone. This study noted an overall PFS-6 of 6% and no difference in median or OS between the two study arms. This is the only study to date to evaluate HU monotherapy for recurrent GBM and suggests that the activity associated with the regimen of imatinib and HU may be primarily related to HU.

Despite the previous single-institutional studies showing that imatinib in addition to HU has modest anti-tumour benefit for patients with recurrent GBM, this regimen had no significant activity in this multi-institutional study that incorporated blinded CIR. The decision to include an increase in corticosteroids as an isolated criterion to define PD in this study remains controversial. Finally, our observation that increased c-KIT copy number may be associated with better outcome suggests that further investigation of the potential prognostic and predictive value of c-KIT may be warranted.

## Figures and Tables

**Table 1:** Patient characteristics at enrolment (ITT population)

		**Study H2201**	**Study H2202**	**All patients**
**Variable**	**Statistic/category**	***N*=131**	***N*=100**	***N*=231**
Age (years)	Median	55.0	56.5	56.0
	Range	18–80	21–75	18–80
Age group, *n* (%)	18–34 years	10 (7.6)	6 (6.0)	16 (6.9)
	35–49 years	31 (23.7)	32 (32)	63 (27.3)
	50–64 years	71 (54.2)	47 (47.0)	118 (51.1)
	⩾65 years	19 (14.5)	15 (15.0)	34 (14.7)
Sex, *n* (%)	Male	79 (60.3)	65 (65.0)	144 (62.3)
	Female	52 (39.7)	35 (35.0)	87 (37.7)
Performance status				
	ECOG 0–1	107 (81.6)	86 (86.0)	193 (83.5)
	ECOG 2	24 (18.3)	14 (14.0)	38 (16.5)
Time since initial diagnosis (months)	Median	9.0	10.0	9.0
	Range	3–45	2–63	3–63
Initial tumour histology, *n* (%)^a^	AA	6 (24.6)	6 (16.0)	12 (5.2)
	GBM	124 (94.7)	94 (94.0)	218 (94.4)
	Gliosarcoma	1 (0.8)	0	1 (0.4)
Time since last recurrence (days)	Median	27.0	26.0	26.0
	Range	1–165	0–222	0–222
Measurable lesions, *n* (%)b,c	0	5 (6.8)	2 (7.1)	7 (6.9)
	1	65 (89.0)	25 (89.3)	90 (89.1)
	2	2 (2.7)	1 (3.6)	3 (3.0)
	>2	1 (1.4)	0 (0.0)	1 (1.0)

a All patients must have independent histological confirmation of their diagnosis as part of their inclusion. This review is still ongoing at the time of the interim analysis.

b Tumour burden measurements are based on central independent review (CIR) data.

c Tumour assessment information was assigned at baseline assessment (first MRI scan). All other data were collected at screening (in some cases this equalled baseline).

**Table 2:** Adverse events in at least 10% of patients (Safety population)

	**Study H2201**	**Study H2202**	**All patients**
	***N*=131**	***N*=99**	***N*=230**
**Adverse event**	***n* (%)**	***n* (%)**	***n* (%)**
*Nausea*			
Grade 3	0	2 (2.0)	2 (0.9)
Grade 4	0	0	0
All grades	50 (38.2)	39 (39.4)	89 (38.7)
			
*Fatigue*			
Grade 3	7 (5.3)	6 (6.1)	13 (5.7)
Grade 4	3 (2.3)	0	3 (1.3)
All grades	41 (31.3)	37 (37.4)	78 (33.9)
			
*Peripheral oedema*			
Grade 3	1 (0.8)	2 (2.0)	3 (1.3)
Grade 4	0	0	0
All grades	31 (23.7)	24 (24.2)	55 (23.9)
			
*Diarrhoea*			
Grade 3	0	1 (1.0)	1 (0.4)
Grade 4	0	0	0
All grades	27 (20.6)	16 (16.2)	43 (18.7)
			
*Thrombocytopaenia*			
Grade 3	8 (6.1)	2 (2.0)	10 (4.3)
Grade 4	4 (3.1)	2 (2.0)	6 (2.6)
All grades	23 (17.6)	11 (11.1)	34 (14.8)
			
*Anaemia*			
Grade 3	5 (3.8)	0	5 (2.2)
Grade 4	0	0	0
All grades	21 (16.0)	11 (11.1)	32 (13.9)
			
*Constipation*			
Grade 3	0	1 (1.0)	1 (0.4)
Grade 4	0	0	0
All grades	22 (16.8)	10 (10.1)	32 (13.9)
			
*Rash*			
Grade 3	0	0	0
Grade 4	0	0	0
All grades	14 (10.7)	13 (13.1)	27 (11.7)

**Table 3:** Pharmacokinetic results

	**AUC (0–24 h) (h ng ml^−1^)**	**AUC (0–12 h) (h ng ml^−1^)**	**AUC (0–tlast) (h ng ml^−1^)**	**AUC (0–tlast) ratio (2)**	**Cmax (ng ml^−1^)**	**Tmax (h)**	***t*_1/2_ (h)**	**CL/F (L h^−^)**	**Lambda_z (1/h)**	**Vz/F (L)**	**Cmax/ dose (1)**	**Cmax ratio (2)**	**AUC/ dose (1)**
Imatinib													
*Study H2201 (w/o EIACDs)*													
Imatinib alone (*N*=15)	49764.9 (49.93)	31984.7 (48.71)			3819.6 (39.81)	3.0 (1–8)	15.017 (43.96)	12.052 (49.90)	0.04618 (44.00)	289.31 (65.97)	6.37 (39.84)		82.94 (49.92)
Imatinib+HU (*N*=10)	57040.1 (37.65)	34610.0 (42.92)			3870.8 (44.88)	4.0 (1–24)	14.465 (13.95)	10.523 (37.68)	0.04791 (13.94)	208.39 (31.60)	6.45 (44.90)		95.09 (37.66)
													
*Study H2202 (with EIACDs)*													
Imatinib alone (*N*=6)	40810.0 (77.47)	24186.3 (77.09)			3062.8 (66.54)	2.5 (2–12)	16.604 (59.84)	24.495 (77.57)	0.04175 (60.14)	567.22 (21.51)	3.06 (66.54)		40.81 (77.47)
Imatinib+HU (*N*=4)	34699.4 (22.55)	17549.2 (30.56)			2284.9 (15.37)	2.5 (2–14)	8.750	28.790 (22.53)	0.07920	280.00	2.28 (15.37)		34.70 (22.55)
													
CGP74588 PK													
*Study H2201*													
Imatinib alone (*N*=15)		5881.1 (56.29)	9806.1 (54.41)	0.1971 (26.52)	678.9 (51.41)	2.0 (1–24)							
Imatinib+HU (*N*=10)		7690.7 (54.87)	13065.7 (52.53)	0.2291 (34.30)	827.6 (54.26)	3.0 (1–4)							
													
*Study H2202*													
Imatinib alone (*N*=6)		8469.3 (77.72)	14388.5 (70.78)	0.3526 (11.59)	1115.5 (65.39)	2.5 (2–12)							
Imatinib+HU (*N*=4)		7310.4 (10.38)	14380.1 (15.26)	0.4145 (16.50)	876.3 (13.84)	2.5 (2–14)							
													
Hydroxyurea													
*Study H2201*													
Imatinib+HU (*N*=10)		51451.5 (50.42)	97642.0 (59.40)		11059.7 (36.47)	7.5 (1–14)	2.928 (15.72)	10.179 (57.71)	0.23615 (15.05)	40.33 (57.62)			
													
*Study H2202*													
Imatinib+HU (*N*=4)		40597.2 (47.04)	85679.3 (25.21)		11597.4 (47.70)	12.5 (1–14)	3.077 (12.99)	11.694 (24.95)	0.22550 (12.38)	49.60 (38.14)			

Abbreviations: EIACDs=enzyme-inducing anticonvulsant drugs; HU=hydroxyurea.

**Table 4:** Tumour marker analysis and progression-free survival

**Variable**	**Result**	**Number of patients**	**Median**	**95% CI**	***P*-value**
*Immunohistochemistry*					
MGMT	Positive	25	37.0	28.0, 75.0	0.68
	Negative	62	49.0	30.0, 55.0	
					
EGFR	Positive	52	50.0	29.0, 55.0	0.083
	Negative	2	26.0	99.0, 43.0	
					
EGFRvIII	Positive	17	49.0	29.0, 57.0	0.863
	Negative	73	50.0	30.0, 55.0	
					
PTEN	Positive	27	54.0	28.0, 62.0	0.88
	Negative	54	39.0	30.0, 54.0	
					
S6	Positive	13	54.0	28.0, 56.0	0.579
	Negative	5	49.0	9.0, 57.0	
					
MAPK	Positive	27	31.0	29.0, 54.0	0.467
	Negative	10	41.0	28.0,56.0	
					
AKT	Positive	24	43.0	29.0, 55.0	0.983
	Negative	16	31.0	29.0, 57.0	
					
VEGF	Positive	39	39.0	29.0, 55.0	0.409
	Negative	25	55.0	50.0, 83.0	
					
PDGFR*α*	Positive	80	49.0	31, 55.0	0.26
	Negative	11	55.0	28.0, 232.0	
					
PDGFR*β*	Positive	47	55.0	37.0, 57.0	0.192
	Negative	20	43.0	29.0, 55.0	
					
*Fluorescence in situ hybridisation*					
EGFR	Increased	13	57.0	49.0, 112.0	0.802
	Normal	19	54.0	29.0, 83.0	
					
PTEN	Deleted	7	54.0	29.0, 135.0	0.804
	Normal	18	55.0	43.0, 83.0	
					
c-KIT	Increased	2	261.0	232.0, 290.0	0.021
	Normal	23	54.0	43.0, 75.0	

Abbreviations: MGMT=methylguanine methyltransferase; PDGFR=platelet-derived growth factor receptor; VEGF=vascular endothelial growth factor.

**Table 5:** Patient outcome

	**Study H2201**	**Study H2202**	**All patients**
	***N*=131**	***N*=100**	***N*=231**
*Analysis population*	*n* (%)	*n* (%)	*n* (%)
ITT population	131 (100)	100 (100)	231 (100)
Safety population	131 (100)	99 (99.0)	230 (99.6)
Per-protocol population	96 (73.3)	71 (71.0)	167 (72.3)
Disease evaluable population	105 (80.2)	80 (80.0)	185 (80.1)
Pharmacokinetic population	15 (11.5)	6 (6.0)	21 (91.0)
			
*Disposition*			
Discontinued study treatment	131 (100)	100 (100)	231 (100)
			
*Main cause of study treatment discontinuation*			
Disease progression	95 (72.5)	73 (73)	168 (72.7)
Death	11 (8.4)	10 (10.0)	21 (9.1)
Adverse event(s)	12 (9.2)	6 (6.0)	18 (7.8)
Subject withdrew consent	5 (3.8)	6 (6.0)	11 (4.8)
Completed prescribed treatment	4 (3.1)	3 (3.0)	7 (3.0)
Administrative problems	2 (1.5)	1 (1.0)	3 (1.3)
Protocol violation	2 (1.5)	0	2 (0.9)
Lost to follow-up	0	1 (1.0)	1 (0.4)
			
*Best overall response, n* (%)			
CR	0	1 (1)	1 (0.4)
PR	5 (3.8)	2 (2.0)	7 (3.0)
SD	26 (19.8)	19 (19.0)	45 (19.5)
Lasting for more than 6 months	8 (6.1)	3 (3.0)	11 (4.8)
PD	79 (60.3)	63 (63.0)	142 (61.5)
			
*Objective response (CR*+*PR), n* (%)	5 (3.8)	3 (3.0)	8 (3.5)
95% confidence interval	1.3–8.7	0.6–8.5	1.5–6.7
			
*Clinical benefit*^a^*, n* (%)	13 (9.9)	6 (6.0)	19 (8.2)
95% confidence interval	5.4–16.4	2.2–12.6	5.0–12.5
			
*CR*+*PR*+*SD, n* (%)	31 (23.7)	22 (22.0)	53 (22.9)
95% confidence interval	16.7–31.9	14.3–31.4	17.7–28.9
			
*CR*+*PR, unconfirmed, n* (%)	9 (6.9)	4 (4.0)	13 (5.6)
95% confidence interval	3.2–12.6	1.1–9.9	3.0–9.4
			
*Progression-free survival time (weeks)*			
Median	6.1	4.6	5.6
95% confidence interval	4.1–7.9	4.1–7.9	4.1–7.9
			
*Progression-free survival at 6 months* (%)			
Median	11.2	9.9	10.6
95% confidence interval	5.7–16.6	3.8–15.9	6.5–14.7
			
*PD determination* (%)			
MRI assessment	61 (48.0)	53 (56.4)	46 (50.5)
Neurological examination but not by MRI	17 (13.4)	9 (9.6)	26 (11.8)
Increased steroid use only	23 (18.1)	18 (19.1)	41 (18.6)
Death without previous PD determination	26 (20.5)	14 (14.9)	40 (18.1)
			
*Overall survival time (weeks)*			
Median	25.3	27.1	26.0
95% confidence interval	19.9–33.0	19.9–39.1	21.3–31.3

Abbreviations: CIR=centralised independent review; CR=complete response; ITT=intent-to-treat; PD=progressive disease; PR=partial response; SD=stable disease.

ITT population=Patients who received at least one dose of any of the two study drugs (imatinib, HU).

Safety population=Patients from the ITT population who had at least one post-baseline safety assessment.

Disease evaluable population=Patients in the ITT population without major protocol deviation.

Per-protocol population=Patients in the ITT population with at least 25 days of treatment with either study drug, with a baseline MRI scan, with a post-baseline MRI scan or a record of death or progression, and without major protocol deviation.

Patients alive and without progression were considered as censored at time of last available tumour assessment.

Progression-free survival time percentiles and rates were calculated by Kaplan–Meier method.

a Clinical benefit was defined as objective response or as SD lasting for >6 months from start of treatment.
