# Supplementary material for: Multicentre phase II studies evaluating imatinib plus hydroxyurea in patients with progressive glioblastoma
Source: Br J Cancer. 2009 Nov 10;101(12):1995–2004. doi: 10.1038/sj.bjc.6605411 (PMC2795431; doi:10.1038/sj.bjc.6605411)
Supplement: Supplementary Methods [file 6605411x1.doc]

**Supplemental Methods: Tumor Biomarker Analaysis**

**Immunohistochemistry**

For all immunohistochemical assays, 5µm sections were cut from paraffin-embedded, formalin-fixed brain tissue, placed on silanized slides, deparaffinized with a series of xylenes, cleared in graded alcohols, and rehydrated. Endogenous peroxidase was quenched using 0.3% H_2_O_2_. Following antigen retrieval, slides were washed in TBS with 0.1% Tween 20 and non-specific protein binding was blocked with 5% normal goat serum for 15 minutes at room temperature. Antibody specifications and detection methods are given in the table below. Nuclear counterstaining was performed using Harris’ modified hematoxylin. The intensity of cytoplasmic/membranous staining detected by IHC was scored on a scale of 0-3+ and the distribution was defined as the percentage of cells with any level of expression.

Intensity scoring of the tumor cells was based on comparison against the internal positive controls, the endothelial cells, which are expected to express the proteins at a level of 2+. If the tumor cells’ intensity of reactivity is equivalent, the case was considered 2, while more intense or less intense staining were considered either 3+ or 0-1+ respectively(Mandell, JW et al. 1998; McLendon, RE et al. 1998; McLendon, RE et al. 2000; Choe, G et al. 2003; Haberler, C et al. 2006; McLendon, RE et al. 2007; Sathornsumetee, S et al. 2008).

| **Assay** | **Antibody Specifications** | **Titration and Incubation of Antibody** | **Antigen Retrieval** | **Detection** | **Interpretation** |
| --- | --- | --- | --- | --- | --- |
| Phospho-S6 | rabbit polyclonal phosphorylated-S6 ribosomal protein (Ser235/236) | phospho-S6 ribosomal protein 1:100 1 hour at room temperature, | 10mM EDTA in decloaker for 5 min at 120ºC | ABC Elite kit α | >20% - positive |
| Phospho-AKT | rabbit polyclonal phosphorylated -AKT (Ser473) (Cell Signaling Technology, Boston, MA) | phospho-AKT 1:50 overnight at 4°C, | 10mM EDTA in decloaker for 5 min at 120ºC | ABC Elite kit α | >20% - positive |
| Phospho-MAPK | rabbit monoclonal phospho-p44/42 MAPK (Thr202/Tyr204 - clone E10) | phospho-p44/42 MAPK 1:100 overnight at 4°C | Sodium Citrate Buffer (pH 6.0) in steamer for 20 min at 95ºC | Multilink Detection Kit β | >20% - positive |
| PTEN | mouse monoclonal PTEN (clone 6H2.1) (Cascade Bioscience, Inc., Winchester, MA) | PTEN 1:1000 overnight at 4°C | Sodium Citrate Buffer (pH 6.0) in steamer for 20 min at 95ºC | Super Sensitive Detection Kit γ | >70% - positive. |
| EGFr | EGFR PharmDx^TM^ kit (Dako Corporation, Carpinteria, CA) | FDA approved manufacturer’s protocol | Enzyme retrieval per manufacturer’s directions | Dako Autostainer instrument per manufacturer’s directions | 21% of higher of cells staining is considered positive. |
| EGFr V III | EGFR vIII (Mab L8A6, gift of Darell Bigner) | overnight at 4°C | HEIR solution 2 for 40 min | Bond Autostainer | Any cell is found that has positive staining, the case is considered positive. |
| MGMT | anti-MGMT (O6-methylguanine-DNA methyltransferase clone mT3.1 [5 µg/ml]; Chemicon International, Temecula, CA) | overnight at 4° C | Sodium Citrate Buffer (pH 6.0) in steamer for 20 min at 95ºC | Super Sensitive Detection Kit γ | <15% - negative;  >15% = positive. |
| VEGF | mouse monoclonal clone VG1, from Thermo Fisher Scientific - Lab Vision | 1:100 with 1 hr RT | ER2 solution on Bond instrument | Bond Refine HRP | Intensity Score  Absent = 0  Mild = 1  Moderate = 2  Strong = 3  Multiply intensity score above by distribution score (percent of reactive cells in tumor). Immunoreactivity scores of greater than 20 were considered high  (positive). |
| PDGFR-A | platelet derived growth factor  receptor alpha rabbit polyclonal antibody from LabVision | 1:100 with 1 hr RT | Dako Target retrieval solution pH 6.1 in 100 degree waterbath for 20 min | Envision plus on Dako Autostainer | Staining is graded as percent of tumor cells that exhibit positive staining.  > 50% = positive  ≤ 50% = negative |
| PDGFR-B | platelet derived growth factor rabbit polyclonal antibody from LabVision | 1:100 with 1 hr RT | Dako Target retrieval solution pH 6.1 in 100 degree waterbath for 20 min | Envision plus on Dako Autostainer | Staining is graded as percent of tumor cells that exhibit positive staining.  > 50% = positive  ≤ 50% = negative |

α 30 minute incubation with goat anti-rabbit secondary; detection with ABC Elite kit (Vector laboratories, Burlingame, CA).

β 30 minute incubation with goat anti-rabbit secondary antibody is followed by detection with the Multilink Detection Kit (Biogenex, San Ramon, CA).

γ 30 minute incubation with goat anti-mouse super sensitive link is followed by detection with Super Sensitive Detection Kit (Biogenex, San Ramon, CA).

# Fluorescence In-Situ Hybridization

Dual-color fluorescent in-situ hybridization (FISH) was performed on formalin-fixed, paraffin-embedded tissue specimens using commercially available probes including EGFR / CEP 7, PTEN/CEP 10 (Vysis, Downers Grove, IL) and c-KIT/CEP 4 for EGFR, PTEN and c-KIT DNA locus copy number, respectively.

Briefly, 5µm sections were cut from paraffin-embedded, formalin-fixed brain tissue and placed on silanized slides. Slides were deparaffinized using xylene and then were dehydrated and cleared of xylene using 100% ethanol. Slides were pretreated with 0.2N HCl at room temperature for 20 minutes then washed in deionized water and 2xSSC for 3 minutes each. Slides were then placed in Pretreatment Solution (Vysis, Downers Grove, IL) at 80ºC for 30 minutes, then washed with two changes of 2xSSC for 5 minutes each. Sections were subjected to digestion with protease at 37ºC for 20-23 minutes, depending upon size of the section. Slides were washed in 2 changes of 2xSSC for 5 minutes each and air-dried. Slides were denatured in a 70% Formamide / 2xSSC solution at 72ºC for 5 minutes and immediately dehydrated in 70%, 85%, and 100% ethanol for 1 minute each. Subsequently, the probe was denatured at 75ºC for 5 minutes, then applied to each slide, sealed with rubber cement, and then placed in a humidified chamber at 37ºC for overnight incubation. After overnight incubation, slides were then washed in 2xSSC / 0.3% NP-40 at room temperature to remove coverslip and then at 72ºC for 2 minutes. DAPI counterstain and a coverslip were applied to the hybridization area. Slides were viewed using an Olympus BX-60 fluorescent microscope. The number of green and orange signals was enumerated in 100 intact, non-overlapping nuclei per slide.

For FISH studies, the cutoff value for chromosomal gain was set at 20%, meaning that greater than 20% of the enumerated nuclei must show greater than two copies of the respective probe. For chromosomal loss, the cutoff value was set at 30% for definitive loss and 20-30% for indeterminate loss. EGFR gene amplification was defined as an EGFR/chromosome 7 centromere ratio of greater than 2.0. Definitive PTEN loss was defined as tumors in which ≥ 30% of nuclei exhibited < 2 copies of PTEN locus and 2 copies of CEP 2 control. Indeterminate PTEN loss refered to tumors in which 20-30% of enumerated nuclei had < 2 copies of PTEN locus and 2 copies of CEP 2 control.

| **Assay** | **Interpretation** |
| --- | --- |
| 7cep | Polysomy – >2 copies per cell  On a 100 cell count, the % cells with polysomy  <15% - intact  >15% - positive |
| EGFR | Polysomy – >2 copies per cell  Amplification - >4 copies per cell  On a 100 cell count, the % cells with polysomy  <15% - intact  >15% - positive |
| 10 cep | Monosomy - <2 copies per cell  On a 100 cell count, the % cells with monosomy  <30% - intact  >30% - positive |
| PTEN | On a 100 cell count, the % cells with monosomy  <30% - intact  >30% - positive |
| 4 cep | Polysomy – >2 copies per cell  On a 100 cell count, the % cells with polysomy  <15% - intact  >15% - positive |
| C kit | Polysomy – >2 copies per cell  Amplification - >4 copies per cell  On a 100 cell count, the % cells with polysomy  <15% - intact  >15% - positive |

1. Choe, G., S. Horvath, et al. (2003). "Analysis of the phosphatidylinositol 3'-kinase signaling pathway in glioblastoma patients in vivo." *Cancer Res* **63**(11): 2742-6

2. Haberler, C., E. Gelpi, et al. (2006). "Immunohistochemical analysis of platelet-derived growth factor receptor-alpha, -beta, c-kit, c-abl, and arg proteins in glioblastoma: possible implications for patient selection for imatinib mesylate therapy." *J Neurooncol* **76**(2): 105-9

3. Mandell, J. W., I. M. Hussaini, et al. (1998). "In situ visualization of intratumor growth factor signaling: immunohistochemical localization of activated ERK/MAP kinase in glial neoplasms." *Am J Pathol* **153**(5): 1411-23

4. McLendon, R. E., L. Cleveland, et al. (1998). "Immunohistochemical detection of the DNA repair enzyme O6-methylguanine-DNA methyltransferase in formalin-fixed, paraffin-embedded astrocytomas." *Lab Invest* **78**(5): 643-4

5. McLendon, R. E., K. Turner, et al. (2007). "Second messenger systems in human gliomas." *Arch Pathol Lab Med* **131**(10): 1585-90

6. McLendon, R. E., C. J. Wikstrand, et al. (2000). "Glioma-associated antigen expression in oligodendroglial neoplasms. Tenascin and epidermal growth factor receptor." *Journal of Histochemistry & Cytochemistry.* **48**(8): 1103-10

7. Sathornsumetee, S., Y. Cao, et al. (2008). "Tumor angiogenic and hypoxic profiles predict radiographic response and survival in malignant astrocytoma patients treated with bevacizumab and irinotecan." *J Clin Oncol* **26**(2): 271-8
